# Fatal Errors

**Published:** 1881-02

**Authors:** Ad. Lippe

**Affiliations:** Phila.


### FATAL ERRORS.

BY AD. LIPPE, M.D., PHILA.

Gonzalvo C. Smythe, M.D., in his work on "Medical Herecies," commits a fatal error in the last sentence of his otherwise very welcome book. "This kind of homœopathy will not stand the test of recent advances in science." The only test of the kind of homœopathy here alluded to, the homœopathy taught by Hahnemann, are the "results." Experiment has clearly shown that the results, the absolute cures obtained, solely depend on a strict adherence to the principles governing the healing art of Hahnemann, called Homœopathy. The departures from these principles have steadily augmented, have really left nothing save a perfect caricature of Hahnemann's healing art; as these departures become more gross the ability to cu e diminishes. In fact these departures were introduced by the same class of men and for the same reasons in this country as they were advocated in Hahnemann's days in his own native county. Then Hahnemann called these men "pretenders," and indignantly charged them with "laziness;" a deplorable want of knowledge causing just such failures as would follow the application of any other laws for any other purposes where they are not fully understood and conscientiously followed. The International Hahnemann Association, fully convinced that a faithful adherence to Hahnemann's teachings would lead to the best results in healing the sick, this conviction being forced upon them by their own experiments tried by themselves, could no longer remain silent when efforts were made to palm off on the community, a caricature for the real healing art. The fatal error now committed by this learned author has been committed time and again by the "progressive men" in their departures and caricatures; but these men never could show superior results, although repeatedly asked to do so. Recent advances in science have clearly illustrated the correctness of Hahnemann's propositions and of his strictly inductive method. We would call the attention of the learned author, at this time to the discoveries made by Prof. Jaeger by means of his neural-analysis. There is science for you, showing that the sickmaking power of drugs increases in the same ratio as the doses are quantitatively diminished. It would have been a *fatal error* had the members of the International Hahnemann Association separated themselves from The American Institute of Homœopathy. Had they resigned their membership *then* Prof. Smythe would have found two separate bodies, of differing homœopathists. He could not be gratified in this partlcular. There can be but one kind of homœopathist, and they adhere strictly to the principles and rules promulgated by its founder in his "Organon." By remaining members of the American Institute the members of the International Hahnemann Association declare their opposition to prevailing departures. By publishing their own platform and principles, they guard against any possible suspicion of endors ing men who, under the mistaken idea of *freedom of medical opinion and action*, reject all and every law and rule governing the healing art; who declare their right to be guided solely by their own individual judgment in treating the sick; and who while to all intents and purposes advocating eclecticism (see *Hom. Times*, Dec. 1880, page 205) irrationally call themselves, what every sensible person knows they are not, homœopaths.

Another fatal error is now proposed. The enterprising publishers Chas. Robson & Co. 920, Chestnut Street, Phila., have issued a circular with interrogatories to homœopathic physicians for the purpose of publishing a book entitled, "The Homœopathic Physicians and Surgeons of America." It would be strange if Dr. Smythe & Co., after exposing the various departures and showing off the "caricatures," should find the followers of Hahnemann, the adherents of his *strict inductive methods*, permitting their names to be published alongside of men who are homœopaths only in name, thereby endorsing all these various shades of *pretenders* as full-fledged homœopathists. Can that "fatal error" be committed? Is it not also a fatal error if the adherents of Hahnemann's strict inductive methods endorse eclectic journals, such as publish eclectic papers, by writing truly homœopathic papers for them? Would it not be best to publish homœopathic papers "separately?"

It is a *fatal* error to suppose that truth and error can co-exist. In Hahnemann's days were found three distinct medical schools: the allopathic, the antipathic, and the homœopathic. To these were added in later days the eclectic school; it is this school of later origin which claims to have found the means by which truth and error can co-exist, and they call themselves rational physicians. What results they have obtained by this irrational mixing up of incongruous principles have not, as yet, been promulgated. It is a *fatal error* to attempt to clothe our Materia Medica Pura in a physiological and pathological livery. This fatal error appears to have been committed by the late Charles Julius Hempel, and has now been revived by H. R. Arndt, M. D., the author of the first volume of a work entitled "Materia Medica and Therapeutics arranged upon a Physiological and Pathological Basis, etc." The intelligent student of our Materica Medica will undoubtedly frequently find under the recorded pathogenesis of almost any remedy, groups of symptoms resembling groups of symptoms he and others have observed in certain classified diseases, or symptoms observed when certain organs or tissues were supposed to be affected by disease. The intelligent student is supposed to be fully acquainted with the little that is known of physiology and pathology, hence these comparisons come to him naturally. If the intelligent student of our Materia Medica is led astray by false teachings; if he is made to believe that a remedy having caused, by developing its sick-making properties, certain symptoms, or groups of symptoms, analogous to similar symptoms also observed in natural diseases therefore becoming a specific remedy in that very form of disease, he commits a fatal error. As an example we will here only call attention to the many chest symptoms caused by *borax*, which resemble pneumonia, in a much higher degree than any other proved remedy; also to the fact that it is but very seldom, a curative remedy for pneumonia, and then only when the *characteristic* symptoms of the drug and the *characteristic* symptoms of the sick fully correspond. If such an attempt as we find the author makes, is to be used even as an aid to the student it should be made intelligently. The very grave diseases, for instance, which we find are very frequently characterized by symptoms similar to those caused by *æsculus hippocastanum*, are not even hinted at. They are *tabes dorsalis* and *locomoter ataxia*. The very first knowledge we had of the sick-making power of *æsculus hippoc* was derived from the poisonous effects it had on a large flock of sheep fed during the winter on the horse-chestnut; these all died of *tabes dorsalis*. Does it therefore follow that this remedy will cure all cases of *tabes dorsalis?* While it resembles in its effects on the cerebro-spinal system very much those of *nux vom.* the intelligent student of Materia Medica, of physiology and pathology, will by *comparisons* between the sick-making properties of *nux vom.*, *phosphor*, zino, *nux mosch.* and *æsculus hippoc.*, acquire a clearer idea of the healing properties of either remedy in the above named forms of disease.

It has been asserted that pathology having become an almost exact science since the days of Hahnemann, we might scientifically venture on the forming of a pathological livery for our simple Materia Medica, that it might appear more acceptable in the eyes of our scientific adversaries. This is again a fatal error; we will now illustrate it. The most scientific pathologists, authorities in diagnosis, at intervals of a year, examined a patient (whose case will be reported elsewhere) and found, one and all, an insufficiency of the mitral value: *therefore* he was treated with digitalis, etc., twice he was snatched from the grave by strict homœopathic treatment, twice was the diagnosis rejected. If then the most competent scientists, made such blunders in their diagnosis, how can any observing man propose to base *our* therapeutics on such a fallacious diagnosis; on such hypotheses as all these diagnosticatiens have so frequently been proved to be? Finally this man died: and of what? On post-mortem examination his heart was perfectly sound, and the difficulty of breathing which was *assumed* to be caused by heart disease had been caused by an adhesion of the posterior left lung, remaining so diseased after allopathic treatment for pneumonia many years ago, the upper part being full of tuberculous deposits and cavities. This diseased condition was latent, and no active disease existed in the lungs at the time of his death. The patient died at an advanced age of chronic hypertrophy of the liver, of which he had suffered many years. So much for the pathology of the present having become an exact science! To put such a livery, such a hypothesis on our Materia Medica is next to madness. Hahnemann was right first and last, and will be right forever. He will be found to have been right just in the same ratio as exact sciences develop the truths broached first by that great observer and philosopher.

